# Multi-Scale Deformable Transformer with Iterative Query Refinement for Hot-Rolled Steel Surface Defect Detection

**DOI:** 10.3390/s25226890

**Published:** 2025-11-11

**Authors:** Haoran Wang, Fan Zhang, Rong Yi

**Affiliations:** 1 College of Mechanical Engineering, University of South China, Hengyang 421001, China; wanghaoran@usc.edu.cn; 2 School of Automation, State Key Laboratory of Precision Manufacturing for Extreme Service Performance, Central South University, Changsha 410083, China; zhangfan219@csu.edu.cn

**Keywords:** surface defect detection, deformable attention, swin transformer, steel surface defects

## Abstract

Accurate and efficient detection of small and complex surface defects on hot-rolled steel plates remains a significant challenge in industrial quality assurance. Current deep learning detectors often exhibit limitations in detection accuracy and training convergence speed, particularly for small objects, which limits their practical deployment in real-time industrial inspection systems. To overcome these deficiencies, this paper proposes a multi-scale deformable transformer iterative query refinement network (MDT-Net). MDT integrates three key innovations: a Swin Transformer backbone for robust multi-scale feature representation, a deformable attention mechanism to significantly reduce computational complexity and accelerate convergence, and an iterative bounding box refinement strategy for precise localization. Extensive experiments on the NEU-DET dataset demonstrate MDT’s superior performance, achieving 82.7% *mAP*_50_. Crucially, MDT significantly outperforms other mainstream detectors in small object detection, reaching an *mAP*_50:95_ of 0.55, and exhibits remarkably faster training convergence. These findings confirm MDT’s effectiveness and robustness for accurate and efficient steel surface defect detection, thereby providing a crucial tool for enhancing sensor-based quality control and offering a promising solution for industrial quality management.

## 1. Introduction

The steel industry is a crucial component of modern industrial production [1]; however, in the actual production process, constrained by manufacturing processes and technology, the surface inevitably develops various defects [2]. For instance, common defects in hot-rolled steel plates include pitting, scratches, holes, scabs, cracks, a pressed-in oxide scale, rust spots, roll marks, flashes, edge cracks, oxidation discoloration, and inclusions [3]. Traditionally, steel surface defect identification [4] was performed using a combination of manual inspection and traditional machine vision methods. However, these methods lack a learning process [5,6], impose stringent requirements on the detection environment, demand the manual selection of high-quality features, and are not suitable for open operating environments. They require specific improvements based on on-site conditions, making generalization difficult [7]. With the widespread application of Convolutional Neural Networks (CNNs) in various industrial image classification and recognition tasks, deep learning-based object detection methods have also gradually found application in a wide variety of surface defect detection tasks [8,9,10,11,12]. For instance, models like the Single-Shot MultiBox Detector (SSD) [13], You Only Look Once (YOLO) [14], Region-based Convolutional Neural Network (Faster R-CNN) [15], and Mask R-CNN [16] have demonstrated impressive performance within the domain of object recognition. Wang et al. [17] designed a Faster R-CNN algorithm that integrated multi-level features, thereby solving the problem of detecting various random defects on metal plates and strips. Dai et al. [18] proposed an efficient algorithm that employed an enhanced Faster R-CNN. This algorithm addresses the limitations and poor precision of part-surface defect detection, significantly enhancing its performance compared to conventional techniques. Zhang et al. [19] boosted the detection performance for small targets in steel plate surface defect detection tasks by modifying the YOLOv3 network’s structure. Nicolas et al. [20] utilized the Detection Transformer (DETR) network structure to achieve end-to-end object detection within the field of computer vision, greatly simplifying the object detection process.

However, these object detection algorithms still result in missed detections and false positives for small targets in surface defect detection tasks. Consequently, we designed a novel method for detecting surface defects using a modified DETR algorithm. By introducing a Swin Transformer as the backbone network, deformable attention, and iterative bounding box refinement, it aims to effectively address the limitations of traditional DETR models’ training efficiency, detection accuracy, and particularly their ability to detect small targets, thereby enhancing the detection precision of surface defects.

The MDT-Net contributions in this study are presented as follows:

(1) Utilizing Swin Transformer as the feature extraction backbone network significantly expands the model’s capacity to capture multi-scale features, effectively acquiring both local and global contextual information from steel plate images.

(2) Introducing deformable attention, which replaces conventional multi-head attention with sampling-based deformable attention, reduces computational complexity and significantly accelerates convergence speed. Concurrently, iterative bounding box refinement improves the localization precision and accuracy of the model.

(3) Using the NEU-DET dataset, the proposed MDT-Net was tested to verify the network’s accuracy in steel plate surface defect detection and to assess the individual and synergistic contributions of Swin Transformer, deformable attention, and iterative bounding box refinement strategies to its performance. The results show an *mAP*_50_ of up to 82.7%, with a significant improvement in small target detection capability.

The remainder of this paper is structured as follows. Section 2, ‘Methods’, details the proposed multi-scale deformable transformer iterative query refinement network (MDT-Net) and its core components. Section 3, ‘Experiments’, presents the experimental setup, results, ablation studies, and a comparative analysis with state-of-the-art object detection algorithms. Finally, Section 4, ‘Conclusions’, summarizes the key findings and contributions of this work.

## 2. Method

### 2.1. Network Architecture Overview

**Figure 1:**
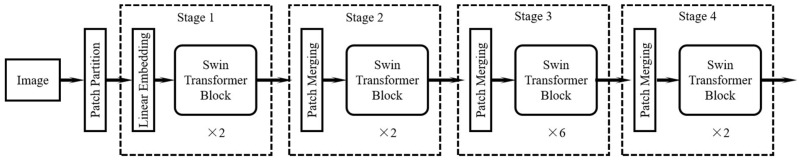
Multi-scale deformable transformer iterative query refinement network.

The architecture of the multi-scale deformable transformer iterative query refinement network is shown in Figure 1. Assuming that the input steel plate image has dimensions *H × W ×* 3 (*H* for height, *W* for width), the image was first divided into several 4 × 4-pixel patches. Subsequently, these image patches underwent a linear embedding operation, which projects the features of each patch into a high-dimensional space and expands them along the channel direction. At this point, the image size is $\frac{H}{4}\times \frac{W}{4}\times 48$. The main body of the network consists of 4 consecutive stages (Stage 1 to Stage 4). Each stage comprises an operation and several transformer block units that utilize deformable self-attention computation. Except for Stage 1, which employs a linear embedding operation, all other stages use block combination operations. After the patched image undergoes linear embedding, its size is $\frac{H}{4}\times \frac{W}{4}\times C$, where *C* is a hyperparameter, and is 96 in the Swin-Tiny architecture. Subsequently, after each stage, the image’s width and height are both half of their original size, and the number of channels doubles. This part of the operation is very similar to a convolutional hierarchical structure.

**Figure 2:**
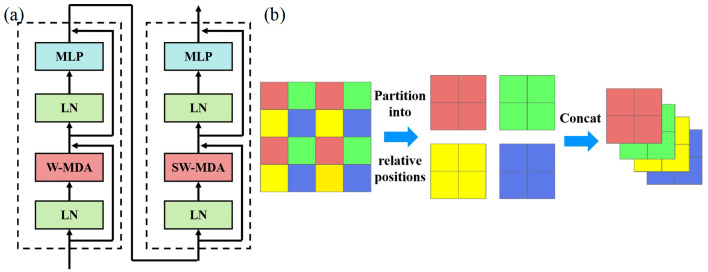
(**a**) Architecture of two consecutive Swin Transformer blocks; (**b**) schematic diagram of the window combination process.

Figure 2a shows the Swin Transformer unit structure. It is largely consistent with the original transformer unit structure; however, the primary distinction lies in the multi-head attention mechanism, which is replaced by window-based multi-head deformable attention (W-MDA) and shifted window-based multi-head deformable attention (SW-MDA). Furthermore, the Multi-Layer Perceptron, which serves as the feed-forward network, comprises two layers, interspersed with a GELU activation function. Notably, Swin Transformer units are utilized in pairs. In this pairing, the attention mechanism within the first unit is based on fixed-window multi-head attention, while the second unit employs a shifted-window multi-head attention. Additionally, the patch merging operation is functionally similar to pooling, aiming to reduce image resolution for downsampling. However, unlike traditional pooling, the mechanism of patch merging involves combining feature values from the same relative positions within a window to form new feature patches and subsequently concatenating these new patches.

### 2.2. Window Multi-Head Attention

The input image is first divided into a series of non-overlapping fixed-size windows. Then, standard multi-head attention is performed in parallel within each independent window. For instance, assuming that the initial input image size is *hw × C*, after applying fixed-window multi-head attention, the input dimensions transform to $\left(\frac{hw}{M^{2}}\right)\times M^{2}\times C$. This means that each individual window has a size of $M^{2}\times C$, and there is a total of $\frac{hw}{M^{2}}$ such windows. The window multi-head attention mechanism is presented as Equation (1).

(1) $$\Omega \left(W-MSA\right)=\left(\frac{hw}{M^{2}}\right)\cdot \left(4M^{2}C^{2}+2M^{4}C\right)=4hwC^{2}+2M^{2}hwC$$

Its time complexity is O(hw), which exhibits an approximately linear relationship with the image size.

### 2.3. Shifted-Window Multi-Head Deformable Attention

**Figure 3:**
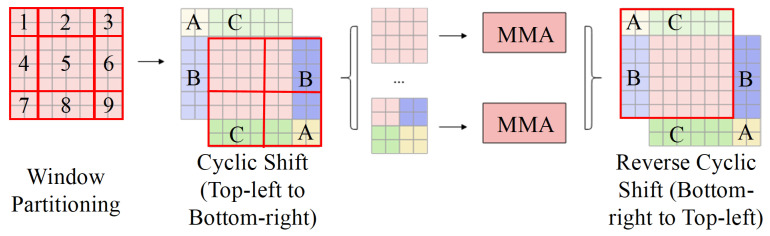
Schematic diagram of Cyclic Shift and Mask Mechanism.

While window multi-head attention reduces computational complexity and overhead, it inherently sacrifices global communication capability. To address this limitation, the shifted-window approach is introduced. Its fundamental process, as depicted in Figure 3, utilizes a Cyclic Shift and Mask Mechanism. This mechanism is crucial for resolving the challenges introduced by sliding windows, specifically the increased number of windows (leading to higher computational complexity) and the inconsistent window sizes (which impede unified parallel computation).

### 2.4. Deformable Attention Mechanism

**Figure 4:**
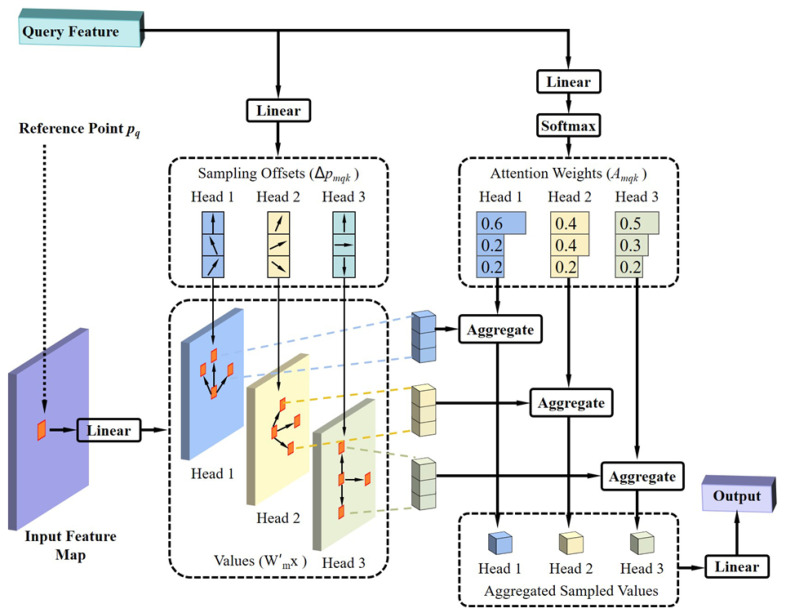
Illustration of the deformable attention module.

The deformable attention mechanism, as depicted in Figure 4, allows each pixel to compute attention solely with a subset of sampled pixels, rather than interacting with every pixel in the entire image. Consequently, this mechanism substantially reduces the computational burden and accelerates the model’s convergence speed. Furthermore, deformable attention concurrently incorporates the principle of multi-scale features. Utilizing deformable attention on multi-scale feature maps can also improve small object detection accuracy, eliminating the need for Feature Pyramid Networks (FPNs) [21]. Equation (2) provides the mathematical formulation of deformable attention.

(2) $$DeformAttn\left(z_{q},p_{q},x\right)=\sum_{m=1}^{M}W_{m}\left[\sum_{k=1}^{K}A_{mqk}\cdot W_{m}^{\prime }x\left(p_{q}+∆p_{mqk}\right)\right]$$

It should provide a concise and precise description of the experimental results, their interpretation, and the experimental conclusions that can be drawn.

Here, reference points represent the 2D coordinates of $z_{q}$ within the feature map. Furthermore, $∆p_{mqk}$ denotes the position offset, which is learnable. *m* denotes the index of the attention head, and $W_{m}$, $W_{m}^{\prime }$ are learnable weight matrices. Each query computes attention solely with the sampled elements within each attention head. Crucially, both $A_{mqk}$ and $∆p_{mqk}$ are derived from query via a fully connected layer, unlike in a standard transformer where they are typically computed from both query and key.

Equation (3) presents the mathematical formulation of multi-scale deformable attention.

(3) $$MSDeformAttn\left(z_{q},{\hat{p}}_{q},{\left\{x^{l}\right\}}_{l=1}^{L}\right)=\sum_{m=1}^{M}W_{m}\left[\sum_{l=1}^{L}\sum_{k=1}^{K}A_{mqk}\cdot W_{m}^{\prime }x^{l}\left(\phi_{l}\left({\hat{p}}_{q}\right)+∆p_{mlqk}\right)\right]$$

Here, the *l*-th multi-scale feature layer is denoted with a total of L layers. Furthermore, ${\hat{p}}_{q}\in \left[0,1\right]$ signifies the normalized reference points. Moreover, $\phi_{l}$ is a function that maps the normalized coordinates to their respective feature layers. Consequently, each reference point possesses a distinct coordinate within each feature layer, thereby facilitating the computation of sampling point locations across these diverse feature layers. In addition, $A_{mqk}$ represents the normalized attention weight. Specifically, each query samples *K* points from each of the *L* feature layers; thus, a total of *KL* pixels were sampled within each attention head. The normalization of attention weights was also conducted over these *KL* pixels, meaning that $\sum_{l=1}^{L}\sum_{k=1}^{K}A_{mqk}=1$.

### 2.5. Iterative Bounding Box Refinement

For each layer of the transformer decoder, predictions are made. These prediction results then serve as new reference points for the subsequent layer, thereby enabling continuous refinement. Ultimately, the final layer’s output is taken as the ultimate result. During its computational process, a regression bounding box detection head is first employed to predict the decoded features, which yields offset results. These offsets are then added to the reference point coordinates and subsequently provided to the next layer. Concurrently, gradients are detached before being fed into the next layer, as the reference points serve as a form of prior information across all decoder layers. For the *d*-th decoder layer, the corrected regression box is given by Equation (4).

(4) $${\hat{b}}_{q}^{d}=\left\{\begin{array}{l} \sigma \left(∆b_{qx}^{d}+\sigma^{-1}\left({\hat{b}}_{qx}^{d-1}\right)\right),\sigma \left(∆b_{qy}^{d}+\sigma^{-1}\left({\hat{b}}_{qy}^{d-1}\right)\right)\\ \sigma \left(∆b_{qw}^{d}+\sigma^{-1}\left({\hat{b}}_{qw}^{d-1}\right)\right),\sigma \left(∆b_{qh}^{d}+\sigma^{-1}\left({\hat{b}}_{qh}^{d-1}\right)\right) \end{array}\right\}$$

(5) $$\sigma =\frac{1}{1+e^{-x}}$$

(6) $$\sigma^{-1}=\log\frac{x}{1-x}$$

Here, $∆b_{q\left(x,y,w,h\right)}^{d}$ represents the prediction result from the regression branch of the *d*-th decoder layer’s output, obtained through the regression bounding box detection head. Furthermore, σ and $\sigma^{-1}$ denote the sigmoid function and its inverse (logit function), respectively. Their mathematical forms are presented in Equations (5) and (6).

### 2.6. Loss Function

MDT-Net adopts the Detection Transformer’s loss function strategy. The model outputs a fixed number of predictions (e.g., 100), which typically exceeds the actual number of ground truth objects. This creates a class imbalance, with many predictions corresponding to no object or background. To address this, a special label (∅) denotes no object or background. When calculating the overall loss ($L_{Hungarian}$), if a predicted box is matched via bipartite matching (Hungarian algorithm) to a $c_{i}$=∅ label (i.e., background class), the logarithmic part of its classification loss is down-weighted by dividing it by 10. This effectively reduces the background class’s contribution to the total loss, mitigating the class imbalance and allowing the model to focus more on detecting actual targets.

(7) $$L_{Hungarian}(y,\hat{y})=\sum_{i=1}^{100}\left[-\log{\hat{p}}_{\hat{\sigma }(i)}(c_{i})+1_{\left\{c_{i}\neq \emptyset \right\}}L_{box}(b_{i},{\hat{b}}_{\hat{\sigma }(i)})\right]$$

Here, y denotes the ground truth, and $\hat{y}$ also represents the 100 predicted boxes. $c_{i}$ is the class of the *i*-th ground truth, and $b_{i}$ denotes the relative values of the bounding box center, width, and height. For the *i*-th prediction $\hat{\sigma }(i)$, the probability of predicting $c_{i}$ is defined as ${\hat{p}}_{\hat{\sigma }(i)}(c_{i})$, and the predicted box is ${\hat{b}}_{\hat{\sigma }(i)}$.

## 3. Experimental Section

### 3.1. Datasets and Evaluation Metrics

**Figure 5:**
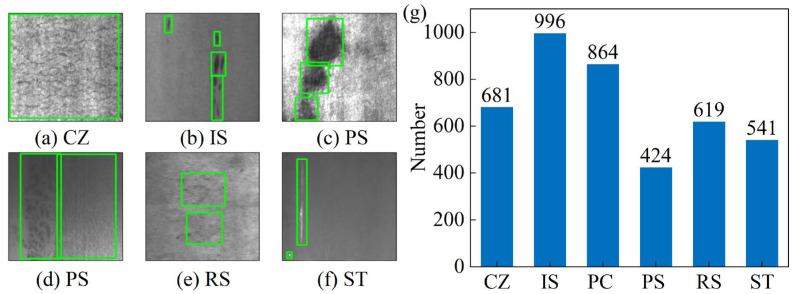
(**a**–**f**) Examples of various defect categories from the NEU-DET dataset, and (**g**) the statistical count of ground truth instances for each defect category.

This study utilizes the NEU-DET hot-rolled steel strip surface defect dataset [22], an open-source resource from Northeastern University. Specifically, this dataset includes 1800 grayscale images, uniformly sized at 200 × 200 pixels. Furthermore, it includes six common defect types present on hot-rolled steel surfaces, which are crazing (CZ), inclusion (IS), pitted surface (PS), patches (PC), scratches (ST), and rolled-in scale (RS). Individual defect images and their corresponding bounding boxes are illustrated in Figure 5a. Given that each image may contain multiple defect types, a statistical analysis was performed on all labeled data to ascertain the actual counts for each defect category. The resulting statistical chart is presented in Figure 5b. The original labels within the dataset are stored in YOLO format, where each bounding box is defined by five values: the class ID, normalized width, normalized height, normalized center x-coordinate, and y-coordinate.

Given that the surface defect dataset comprises only 1800 images, offline augmentation was initially performed to expand the dataset size. Specifically, this experiment employed random rotation, scaling to 400 × 400 pixels, and random cropping, thereby increasing the training images to 9000. For training, the dataset was split 9:1 into training and validation sets. The experiments utilized the PyTorch-based deep learning framework MMDetection. The Swin Transformer was chosen as the Swin-Tiny model, and it utilized pre-trained weights from ImageNet-1K. The embedding dimension was set to 96, while the number of Swin Transformer units for each stage amounted to two, two, six, and two, respectively. The multi-head attention heads for each stage were 3, 6, 12, and 24, with a window size of seven and a patch size (pixel) of four. The linear embedding/merging strides were four, two, two, and two.

The main parameters for the deformable attention-based transformer are as follows: 300 object queries, 3 encoder hidden layers, a Dropout probability of 0.1 for the feed-forward network in each encoder layer, 3 decoder hidden layers, a 0.1 Dropout probability for the feed-forward network in each decoder layer, and 4 multi-head attention heads in the decoder. The AdamW (Adam with decoupled Weight decay) optimizer was employed, with a 0.0002 learning rate and a 0.0001 weight decay. In addition, the number of iterations, batch size, and number of workers were 100, 8, and 2, respectively.

This paper adopts the metric of *IoU* > 0.5 as the accuracy indicator for surface defect detection, denoted as *mAP*_50_. Mathematically, its meaning is the ratio of the area under the precision (P)–recall (R) curve to the total area. Specifically, precision and recall are defined by Equations (8) and (9) [23].

(8) $$P=\frac{TP}{TP+FP}$$

(9) $$R=\frac{TP}{TP+FN}$$

(10) $$AP=\int P\left(R\right)dR$$

(11) $$mAP=\frac{\sum_{i=1}^{N}AP}{TN}$$

Here, *TP*, *FP*, and *FN* represent the number of correctly detected, incorrectly detected, and missed targets for comparative experiments; to validate the enhanced capability of the proposed model for small object detection, the *mAP*_50:95_ metric was also utilized. Specifically, this metric is derived by considering 10 distinct *IoU* thresholds (from 0.5 to 0.95 at 0.05 intervals). For each of these *IoU* thresholds, an individual mean average precision (*mAP*) value is computed, and *mAP*_50:95_ is defined as the average of these 10 values.

### 3.2. Experimental Results

**Figure 6:**
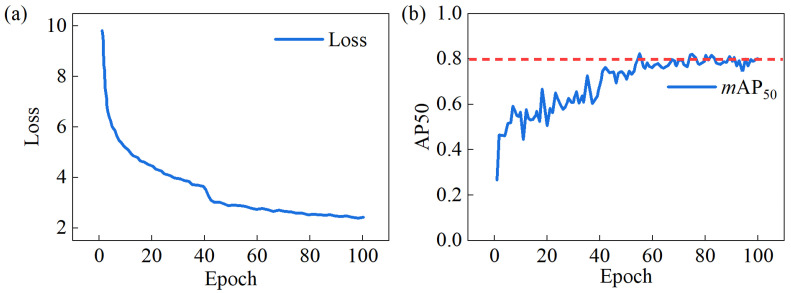
(**a**) Loss curve and (**b**) *mAP*_50_ curve.

The training process’s *loss* curve and *mAP*_50_ curve are both depicted in Figure 6. Around the 40th epoch, the *loss* curve showed a sudden decrease, while the *mAP*_50_ curve exhibited a sharp increase. This phenomenon occurred because the training strategy adjusted the learning rate at the 40th epoch, reducing it tenfold from 0.0002 to 0.00002. After 100 iterations, the optimal *mAP*_50_ value reached 82.7%. The *mAP*_50_ for each defect category is as follows: crazing, 50.2%; inclusion, 87.4%; patches, 92.7%; pitted surface, 86.0%; rolled-in scale, 83.0%; and scratches, 96.9%. The average value is 82.7%.

**Figure 7:**
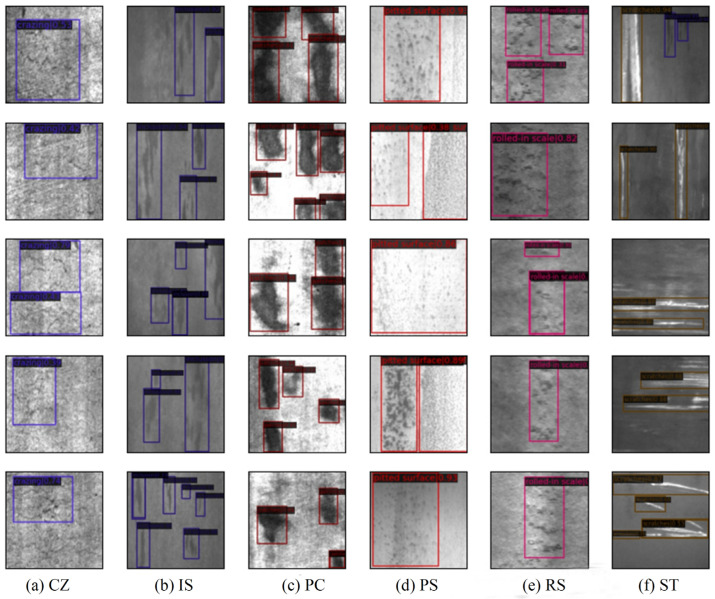
(**a**–**f**) Defect detection results for different categories.

From each category of the output results, five images were selected, as shown in Figure 7. Based on the detection images and the numerical results, it is clear that although the MDT-Net model’s accuracy for detecting crazing defects is relatively low at only 50.2%, the detection accuracy for other defect categories is above 80%, with the highest accuracy for scratches reaching 96.9%. Therefore, the improved algorithm in this study demonstrates strong performance within the domain of steel strip surface defect detection.

### 3.3. Ablation Experiments

In order to systematically evaluate the effectiveness of the MDT-Net algorithm’s key components, we conducted ablation experiments. These experiments aim to quantify the independent and combined contributions of the Swin Transformer backbone network, the deformable attention mechanism, and the iterative bounding box refinement strategy to the steel plate surface defect detection performance. The module configuration information for the five sets of models is shown in Table 1. Experiment 1 serves as the baseline, employing the original DETR algorithm (using ResNet50 as the backbone network). Subsequent experiments progressively introduced or replaced different components.

The ablation experiment results are shown in Table 1. It is clear that Experiment 5 (our algorithm) achieved the highest *mAP*_50_ of 82.7%. Compared to the original DETR baseline (Experiment 1, *mAP*_50_ = 77.0%), the method presented in this paper achieved a significant improvement of 5.7%. This demonstrates that the proposed combined strategy can effectively increase the accuracy of steel plate surface defect detection.

Comparing Experiment 1 and Experiment 2, it is observed that when deformable attention was introduced in isolation, *mAP*_50_ slightly decreased from 77.0% to 76.7%. This suggests that, without modifying the backbone network and in the absence of other optimizations, deformable attention might necessitate more meticulous fine-tuning or integration with a more robust feature extractor to fully realize its benefits. However, when combined with iterative bounding box optimization (Experiment 4 vs. Experiment 2) and the Swin Transformer backbone (Experiment 5 vs. Experiment 3), deformable attention exhibited a synergistic effect in performance enhancement. Replacing the backbone network from ResNet50 with Swin Transformer (Experiment 3 vs. Experiment 1) increased *mAP*_50_ from 77.0% to 77.3%, thereby showcasing the advantage of Swin Transformer in feature extraction. Nevertheless, the Swin Transformer backbone introduced a higher computational load (FLOPs: 26.55 G vs. 15.19 G) and a lower detection speed (21 FPS vs. 28 FPS), which is consistent with the inherent complexity of the Swin Transformer. Iterative bounding box optimization for prediction frames proved crucial for improving performance. Specifically, comparing Experiment 2 and Experiment 4 for the introduction of iterative optimization, in the presence of deformable attention, *mAP*_50_ was significantly increased from 76.7% to 78.6%. Similarly, under the Swin Transformer backbone network (Experiment 5 vs. Experiment 3), iterative optimization led to a substantial jump in *mAP*_50_ from 77.3% to 82.7%, making it a key factor in achieving the final high accuracy. This unequivocally confirms the effectiveness of the iterative correction mechanism in progressively refining the predicted bounding boxes.

**Figure 8:**
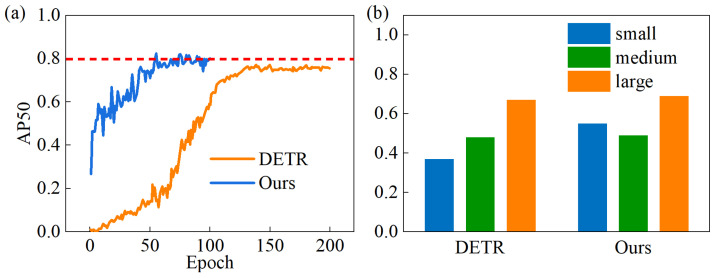
(**a**) *mAP*_50_ curves of our and (**b**) the DETR algorithm.

To further evaluate the performance of the MDT-Net in terms of the training efficiency and detection of different-sized targets, we compared its *mAP*_50_ convergence curve and detection performance for various target sizes against the original DETR algorithm, as illustrated in Figure 8. It can be observed that the MDT-Net model entered the convergence stage around the 55th epoch, whereas the original DETR algorithm only reached convergence around the 130th epoch. This indicates that the MDT-Net model not only significantly accelerated the training convergence speed but also achieved higher detection accuracy after convergence. Figure 8b compares the *mAP*_50_ of the proposed model and the original DETR algorithm for small, medium, and large targets. These results show that the MDT-Net model’s detection capability for small targets is significantly stronger than that of the DETR algorithm, while for medium and large targets, the detection performance of both models is not substantially different. This highlights the advantage of the MDT-Net model in handling common small defects on steel plates, which is crucial for practical industrial applications.

**Figure 9:**
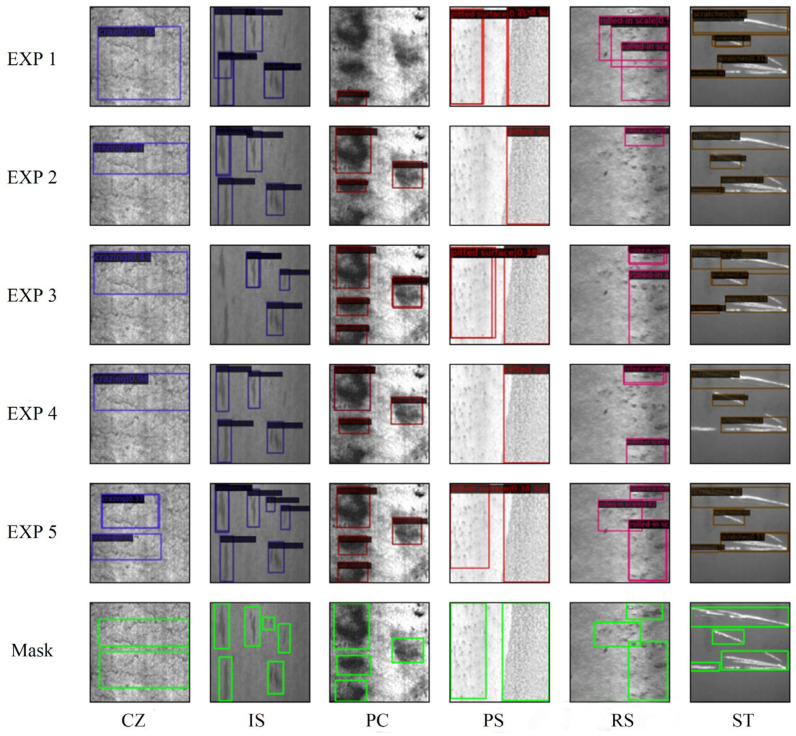
Comparison of steel plate defect detection images from ablation experiment results.

Figure 9 illustrates a visual comparison of the detection results for different defect types across various experimental groups. As can be seen from the figure, Experiment 5 demonstrated significant advantages in terms of localization accuracy and bounding box tightness in detecting defects. In particular, when detecting small-sized or irregularly shaped defects, Experiment 5 was able to generate more accurate prediction boxes and effectively reduced instances of missed detections and false positives. This is highly consistent with the quantitative results presented in Table 1. For subtle scratches or pitted defects under certain complex backgrounds, the detection performance of Experiment 5 was significantly superior compared to that of other experimental groups. The ablation experiments unequivocally confirmed the effectiveness of the Swin Transformer backbone network, the deformable attention mechanism, and the iterative bounding box refinement strategy for prediction. The synergistic effect of these components significantly improved steel plate surface defect detection accuracy. Furthermore, the performance enhancement was more pronounced after the introduction of iterative bounding box refinement.

Table 2 presents an ablation study on the hyperparameters of our deformable attention-based Transformer, revealing their impact on *mAP*_50_. The study identifies an optimal configuration of three encoder layers, three decoder layers, and four attention heads, achieving the highest *mAP*_50_ of 82.7%. Crucially, increasing the number of attention heads from one to four (while keeping layers at 3,3) leads to a substantial performance gain, improving *mAP*_50_ from 79.6% to 82.7%, and underscoring the importance of diverse feature learning. Conversely, varying the encoder and decoder hidden layers (with four heads) shows that both shallower (2,2) and deeper (4,4) architectures yield slightly lower performances (80.6% and 81.3%, respectively) compared to the optimal (3,3) configuration, suggesting a sweet spot for network depth. This systematic evaluation highlights the critical role of careful hyperparameter tuning in achieving optimal accuracy for steel surface defect detection.

### 3.4. Algorithm Comparison

To validate the superiority of the MDT-Net model over other models, comparative experiments utilized the NEU-DET dataset. Specifically, YOLOv3, SSD, and Faster R-CNN models were selected for comparison, resulting in a total of four models, including the one proposed in this paper. The *mAP*_50_ results for the YOLOv3, SSD, Faster R-CNN, and the MDT-Net model were 79.6%, 76.1%, 79.4%, and 82.7%, respectively. This indicates MDT-Net’s superior overall performance in steel plate surface defect detection.

**Figure 10:**
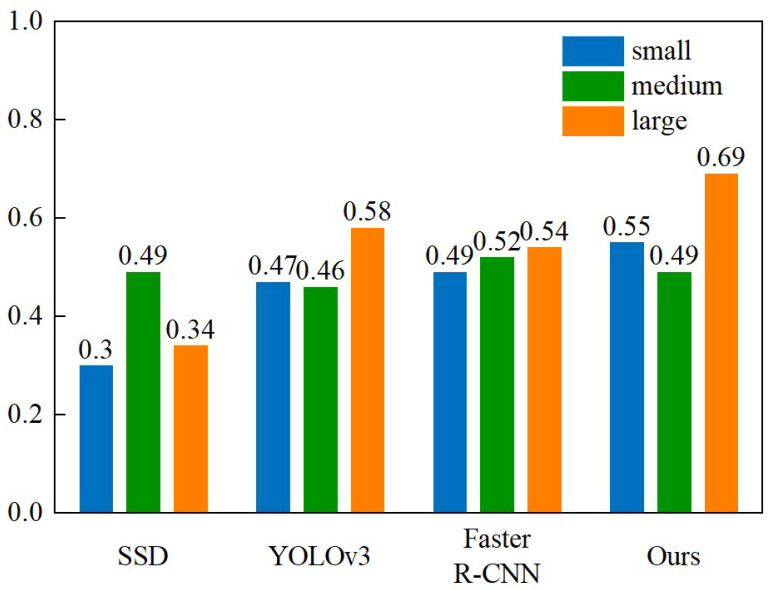
Comparison of *mAP*_50:95_ for SSD, YOLOv3, Faster R-CNN, and our object detection algorithm across three sizes.

To further analyze the MDT-Net detection capabilities for defects of different scales, we additionally compared the *mAP*_50:95_ of each algorithm for small, medium, and large targets, and Figure 10 displays the detailed results. The MDT-Net model achieved an *mAP*_50:95_ of 0.55 for small target detection, which is significantly higher than YOLOv3 (0.47), SSD (0.30), and Faster R-CNN (0.49). This fully demonstrates the remarkable performance of the MDT-Net model in handling small defects commonly found on steel plate surfaces.

**Figure 11:**
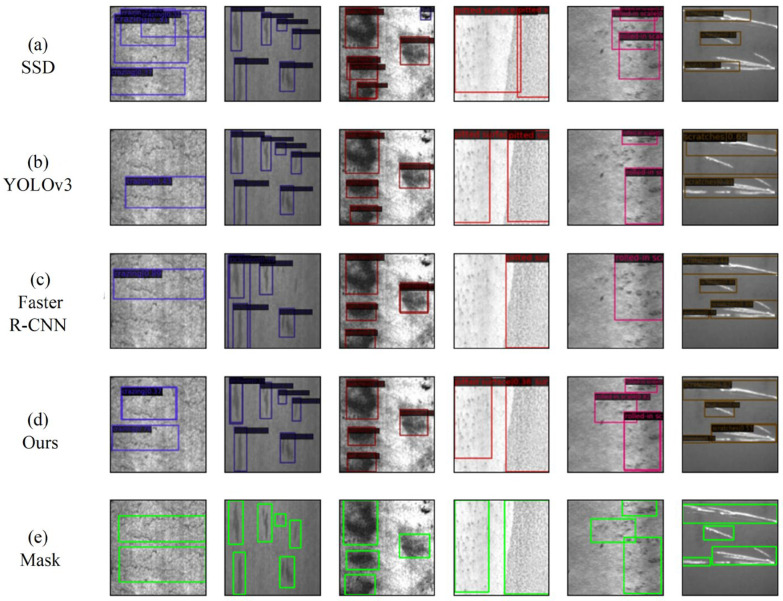
(**a**) SSD, (**b**) YOLOv3, (**c**) Faster R-CNN, (**d**) our method, and (**e**) Mask steel plate defect detection results.

From Figure 11, it is evident that the MDT-Net model exhibits clear advantages in the localization accuracy and bounding box tightness of defects, especially when detecting small-sized or irregularly shaped defects. Particularly in areas with complex backgrounds or dense defects, the proposed model can generate more accurate prediction boxes and effectively reduce missed detections and false positives. This is highly consistent with the significant improvement in small target detection shown in Figure 10.

Table 3, detailing per-category *mAP*_50_ results across various state-of-the-art methods, confirms MDT-Net’s overall superiority in steel plate defect detection, achieving the highest average *mAP*_50_ of 82.7%. Specifically, MDT-Net demonstrates a leading performance in most defect categories, notably achieving the highest accuracy for inclusion (87.4%), patches (92.7%), rolled-in scale (83.0%), and an impressive 96.9% for scratches, significantly surpassing competitors and highlighting its capability with subtle defects. However, while competitive for pitted surface (86.0%), MDT-Net records the lowest *mAP*_50_ for crazing defects at 50.2%, which is consistent with earlier observations and suggests challenges with their irregular and subtle characteristics. Despite this specific challenge, MDT-Net’s robust and often superior performance across other defect types solidifies its position as a highly effective and practical solution for industrial steel plate surface defect detection.

Based on quantitative results and qualitative analysis, the multi-scale deformable transformer with iterative query refinement proposed in this paper demonstrates superior comprehensive performance compared to mainstream object detection algorithms for steel plate surface defect detection on the NEU-DET dataset; it demonstrates particularly significant advantages in small target detection. This further validates the performance of the Swin Transformer backbone network, the deformable attention mechanism, and the iterative bounding box refinement strategy that we introduced, making it a powerful tool for steel plate surface defect detection.

## 4. Conclusions

This paper proposes a novel MDT-Net, aimed at enhancing the accuracy of hot-rolled steel plate defect detection. Specifically, (1) the introduction of Swin Transformer as the backbone network significantly improved the network’s multi-scale feature representation capability, effectively capturing both local and global contextual information from steel plate images. (2) The integration of a deformable attention mechanism substantially reduced computational complexity and accelerated model convergence. By applying deformable attention to multi-scale feature maps separately, the detection capability for small targets was enhanced. (3) Through iterative bounding box optimization, localization accuracy was improved, leading to a significant increase in both defect localization precision and overall detection accuracy for the steel plates. Experimental results demonstrate that this method can effectively boost steel plate surface defect detection accuracy, achieving an *mAP*_50_ of 82.7%. Compared to other models, it exhibits significant advantages in small target detection, thus offering a new approach for steel plate defect detection. Given the NEU-DET dataset’s limitations in size and diversity, future work should prioritize acquiring more complex and varied real-world steel surface defect datasets to enable a robust evaluation of MDT-Net’s practical industrial performance.

## Figures and Tables

**Table 1:** Comparison of data results for ablation experiments.

Schemes	D A	S T	IBBR	*mAP*_50_	Parameters	FLOPs	Detection Speed
1				77.0%	32.6 M	15.19 G	28 FPS
2	√			76.7%	34.49 M	24.67 G	24 FPS
3	√	√		77.3%	33.51 M	26.55 G	21 FPS
4	√		√	78.6%	34.76 M	24.79 G	23 FPS
5	√	√	√	82.7%	33.78 M	26.67 G	18 FPS

**Table 2:** Ablation study of the hyperparameters for the deformable attention-based transformer.

Coder Hidden Layers	Decoder Hidden Layers	Head Number	*mAP*_50_
3	3	1	79.6%
3	3	2	80.3%
3	3	4	82.7%
4	4	4	81.3%
2	2	4	80.6%

**Table 3:** Comparing the proposed method with the other approaches for each type of defect.

Methods	*mAP*_50_	CZ	IS	PC	PS	RS	ST
SSD	76.1%	52.1%	75.3%	86.5%	81.5%	74.1%	87.2%
YOLOv3	79.6%	57.4%	78.4%	88.5%	86.1%	77.5%	89.8%
Faster R-CNN	79.4%	58.6%	76.7%	89.4%	84.7%	76.7%	90.4%
HyperNet	74.8%	54.1%	68.0%	86.5%	87.0%	65.2%	88.1%
Ours	82.7%	50.2%	87.4%	92.7%	86%	83%	96.9%

## Data Availability

The raw data supporting the conclusions of this article will be made available by the authors without undue reservation.
